# Artificial Intelligence Models for Diagnosis of Periodontitis Using Non-Invasive Biological Markers: A Systematic Review and Meta-Analysis of Patient-Based Studies

**DOI:** 10.3390/medsci13030159

**Published:** 2025-09-01

**Authors:** Carlos M. Ardila, Anny M. Vivares-Builes, Pradeep Kumar Yadalam

**Affiliations:** 1 Department of Periodontics, Saveetha Dental College, and Hospitals, Saveetha Institute of Medical and Technical Sciences, Saveetha University, Saveetha 600077, India; pradeepkumar.sdc@saveetha.com; 2 Biomedical Stomatology Research Group, Basic Sciences Department, Faculty of Dentistry, Universidad de Antioquia U de A, Medellín 050010, Colombia; anny.vivares@uam.edu.co; 3 Institución Universitaria Visión de las Américas, Faculty of Dentistry, Medellín 050040, Colombia

**Keywords:** periodontitis, artificial intelligence, machine learning, saliva, biomarkers, diagnostic accuracy, systematic review, meta-analysis

## Abstract

**Background/Objectives**: Early diagnosis of periodontitis remains challenging using traditional clinical methods. This systematic review and meta-analysis evaluated the diagnostic accuracy of artificial intelligence (AI) models trained on non-invasive or minimally invasive biomarkers—including saliva, gingival crevicular fluid (GCF), and immunologic profiles—for diagnosing and classifying periodontitis in human subjects. **Methods**: A comprehensive search of PubMed/MEDLINE, Scopus, Web of Science, EMBASE, and Cochrane CENTRAL was conducted from database inception to June 2025. Eligible studies used AI or machine learning models with patient-derived biomarker data and reported diagnostic performance metrics. **Results**: Seven studies were included, employing various AI models such as random forest, artificial neural networks, and gradient boosting. Biomarkers were derived from saliva (n = 4), saliva-derived biomarkers from oral rinse (n = 1), immunologic profiles (n = 1), and tissue-based gene expression (n = 1). Reported area under the receiver operating characteristic (ROC) curve (AUC) ranged from 0.83 to 0.96. Meta-analysis of studies with comparable outcomes showed a pooled sensitivity of 0.89 (95% CI: 0.84–0.93), a specificity of 0.87 (95% CI: 0.80–0.92), and a summary AUC of 0.92. Subgroup analysis revealed that models using salivary biomarkers achieved a higher pooled AUC (0.94) than those using GCF or immunologic markers (AUC: 0.89). Sensitivity analyses excluding studies with unclear bias did not significantly alter pooled estimates, affirming robustness. The overall certainty of evidence was rated as moderate to high. **Conclusions**: AI-based diagnostic models utilizing salivary, microbiome, or immunologic biomarkers demonstrated quantitatively high accuracy; however, the overall certainty of evidence was rated as moderate to high due to limitations in study design and validation.

## 1. Introduction

Periodontitis is a highly prevalent chronic inflammatory disease characterized by the progressive destruction of the supporting tissues of the teeth. It represents one of the leading causes of tooth loss in adults and has been associated with a wide spectrum of systemic conditions, including diabetes, cardiovascular and respiratory diseases, rheumatoid arthritis, neurological disorders, and certain cancers [1,2]. The global burden of periodontitis remains substantial. Recent meta-analyses report that around 62% of dentate adults had periodontitis between 2011 and 2020, with 24% exhibiting severe forms. In 2021, the global burden of disease (GBD) was estimated to be approximately 951 million cases among individuals aged 15–69, corresponding to an age-standardized prevalence of ~17%, with an estimated 11% of the worldwide population suffering from severe disease. In addition, GBD data suggest that periodontitis affected around 12.5% of the global population in 2021 [3].

Conventional clinical diagnosis of periodontitis is based on defined criteria, including clinical attachment loss at two or more non-adjacent teeth, probing depth ≥ 4 mm, bleeding on probing, and radiographic evidence of alveolar bone loss. However, these indicators reflect past tissue destruction and offer limited value for early detection or prediction of disease progression [4,5]. In response, there is growing interest in non-invasive diagnostic methods with improved sensitivity, specificity, and prognostic potential to enable more timely therapeutic intervention.

In this context, artificial intelligence (AI) and particularly machine learning (ML) models have emerged as promising tools for the non-invasive diagnosis of periodontitis. These models can integrate diverse biological variables—including salivary biomarkers, oral microbiome signatures, and immunological indicators—to automatically predict a patient’s periodontal status. Recent studies have shown that AI models trained on saliva-based features can achieve high diagnostic performance, with area under the curve (AUC) values ranging from 0.93 to 0.96 [4,5,6]. Algorithms such as random forests, artificial neural networks, and Extreme Gradient Boosting have been applied to gene expression data, bacterial copy numbers, or immune profiles, demonstrating robust discriminative capabilities in both binary classification (healthy vs. diseased) and multiclass settings (health, gingivitis, and various stages of periodontitis) [6,7].

Salivary microbiota, in particular, have been widely studied as a precision biomarker for periodontitis. Amplicon sequence variant (ASV) analysis via 16S sequencing has revealed microbial species consistently associated with the disease, although technical confounders such as batch effects challenge the reproducibility and robustness of predictive models [8,9,10]. Furthermore, species-level analyses have shown that even well-maintained periodontitis cases exhibit distinct salivary microbial profiles compared to healthy individuals, suggesting persistent microbial signatures that may indicate susceptibility despite clinical resolution [9]. Alternatively, composite models integrating self-reported data, inflammatory biomarkers such as activated MMP-8, and point-of-care diagnostics have shown high sensitivity and specificity and are proposed as scalable tools for population-level screening [6]. However, variability in sampling sites, sequencing methods, and diagnostic definitions remains a major obstacle to standardization and widespread clinical application of microbial biomarkers [11].

Despite the increasing number of studies exploring AI in periodontal diagnostics, substantial methodological heterogeneity persists—particularly in the choice of biomarkers, model architectures, and performance reporting—which hinders the generalization of findings and limits clinical translation [12,13]. This heterogeneity is evidenced by variable diagnostic targets (e.g., bone loss detection vs. risk prediction), differences in imaging modalities or biomarker inputs, and the inconsistent application of AI algorithms across studies [12]. Furthermore, limitations in study design—such as retrospective approaches, lack of standardized data collection, and underutilization of modern deep learning networks—underscore the need for methodological harmonization to enable broader clinical integration [14]. Moreover, many studies lack external validation or fail to fully report diagnostic metrics, impeding reliable cross-study comparisons [15,16,17].

To address these gaps, the present systematic review and meta-analysis aims to rigorously evaluate the diagnostic performance and clinical utility of AI-based models employing non-invasive or minimally invasive biomarkers—such as salivary, microbiome, gingival crevicular fluid (GCF), or blood-derived immunological profiles—for diagnosing periodontitis in human subjects. Through quantitative and qualitative synthesis, this review seeks to identify the most promising algorithms, assess their translational potential, and outline current limitations that must be overcome to enable effective clinical integration.

## 2. Materials and Methods

This systematic review and meta-analysis was conducted in accordance with the Preferred Reporting Items for Systematic Reviews and Meta-Analyses (PRISMA) 2020 guidelines to ensure transparency and methodological rigor [18]. The protocol was prospectively registered in the International Prospective Register of Systematic Reviews (PROSPERO) under the registration number CRD420251102399.

### 2.1. PIRT Framework

This review was structured according to the PIRT (Population, Index test, Reference standard, Target condition) framework, which is recommended for systematic reviews of diagnostic accuracy to ensure methodological clarity and alignment with PRISMA-DTA (Preferred Reporting Items for Systematic Reviews and Meta-Analyses of Diagnostic Test Accuracy Studies) and QUADAS-2 (Quality Assessment of Diagnostic Accuracy Studies, version 2) guidelines.

Population: Human subjects diagnosed with periodontitis or classified as periodontally healthy, based on established clinical criteria.

Index Test (I): AI- or ML-based diagnostic models trained on non-invasive or minimally invasive biomarker data, including saliva, oral microbiome, gingival crevicular fluid, or immunologic profiles.

Reference Standard (R): Conventional periodontal clinical diagnosis or classification according to the 2017 American Academy of Periodontology (AAP) and European Federation of Periodontology (EFP) case definitions [19].

Target Condition (T): Presence, classification, or staging of periodontitis.

Diagnostic accuracy measures extracted included sensitivity, specificity, area under the ROC curve (AUC), and model performance metrics such as confusion matrices and F1-score (harmonic mean of precision and recall, ranging from 0 to 1), as well as information on internal and external validation.

### 2.2. Eligibility Criteria

Studies were selected based on predefined eligibility criteria to ensure clinical relevance and methodological rigor. Only original articles published in peer-reviewed journals and involving human participants were considered eligible. Included studies were required to utilize biological samples obtained through non-invasive or minimally invasive methods, such as saliva, GCF, peripheral blood, or oral tissue, provided that these samples enabled the extraction of biomarkers relevant for diagnosing or classifying periodontal health status.

Eligible studies had to apply machine learning, artificial intelligence, or advanced statistical models for the development of diagnostic or classification tools targeting periodontal disease. Furthermore, they were required to report measurable diagnostic performance metrics, including AUC, sensitivity, specificity, accuracy, precision, F1-score, or confusion matrices. To ensure clinical applicability, studies were expected to build and/or validate models using data derived from real human subjects, whether through independent cohorts or appropriate internal cross-validation techniques.

Studies were excluded if they involved only in vitro experiments or animal models, or if they relied exclusively on imaging-based approaches (such as radiographs or CBCT) without incorporating biological or biomarker-based data. Likewise, studies based solely on in silico bioinformatic analyses of public databases without patient-level clinical data, or those relying entirely on synthetically generated datasets with no empirical grounding in human-derived samples, were excluded. This approach ensured that the evidence synthesized in the review remained clinically anchored and diagnostically meaningful.

### 2.3. Search Strategy and Information Sources

A comprehensive literature search was conducted across five major databases—PubMed/MEDLINE, Scopus, Web of Science, EMBASE, and Cochrane CENTRAL—from database inception to June 2025. The search strategy incorporated both MeSH terms and keywords relevant to periodontitis, salivary diagnostics, microbiota, biomarkers, and AI/ML techniques. The full electronic search strings for each database, including all terms, Boolean operators, and database-specific adaptations, are provided in Supplementary Table S1. Reference lists of included articles were manually screened to identify additional relevant studies. No date or language restrictions were applied during the search.

### 2.4. Study Selection and Data Extraction

Two reviewers independently performed the screening of titles and abstracts and assessed the full texts of potentially eligible studies. Disagreements were resolved by discussion or consultation with a third reviewer. Data extraction was carried out using a standardized form, capturing details such as study design, participant demographics, sample size, biomarker type, AI model architecture, diagnostic targets (binary or multiclass), validation strategy, and performance metrics.

### 2.5. Data Synthesis and Meta-Analysis

A narrative synthesis was conducted to summarize key study features, biomarker types, algorithms used, validation strategies, and diagnostic targets. Where sufficient homogeneity existed, a meta-analysis was performed using a random-effects model to account for anticipated variability across studies. Pooled estimates for diagnostic accuracy measures (e.g., AUC, sensitivity, specificity) were calculated using logit-transformed values. The DerSimonian–Laird estimator was implemented using Python (version 3.11) with the statsmodels and matplotlib libraries for statistical estimation and forest plot generation.

Statistical heterogeneity among studies was assessed using the I^2^ statistic, which quantifies the proportion of variability due to heterogeneity rather than chance, and τ^2^ (tau-squared), which estimates between-study variance in a random-effects model. When heterogeneity was present (I^2^ > 50%), additional subgroup and sensitivity analyses were performed to explore potential sources, such as biomarker type or machine learning model architecture.

### 2.6. Risk of Bias and Evidence Quality

Risk of bias was assessed independently by two reviewers using an adaptation of the QUADAS-2 tool specifically modified for AI-based diagnostic studies [20]. This adaptation included refinement of the index test domain to evaluate whether AI models were trained using appropriate data partitions (e.g., cross-validation, test splits), whether overfitting was mitigated through proper validation strategies, and whether external validation and transparency of model architecture were reported. The domains evaluated included patient selection, index test development and application, reference standard (clinical diagnosis), and flow and timing.

The certainty of evidence across outcomes was assessed using the Grading of Recommendations, Assessment, Development and Evaluation (GRADE) framework [21]. Despite its original design for intervention studies, GRADE is increasingly used in diagnostic accuracy research, particularly for AI models, as it enables structured evaluation of key factors such as inconsistency in diagnostic performance across studies, indirectness of data sources or population, and imprecision in performance metrics like AUC or sensitivity. It was deemed appropriate in this context to evaluate the overall confidence in the evidence by considering risk of bias, inconsistency, indirectness, imprecision, and publication bias.

## 3. Results

### 3.1. Study Selection

**Figure 1:**
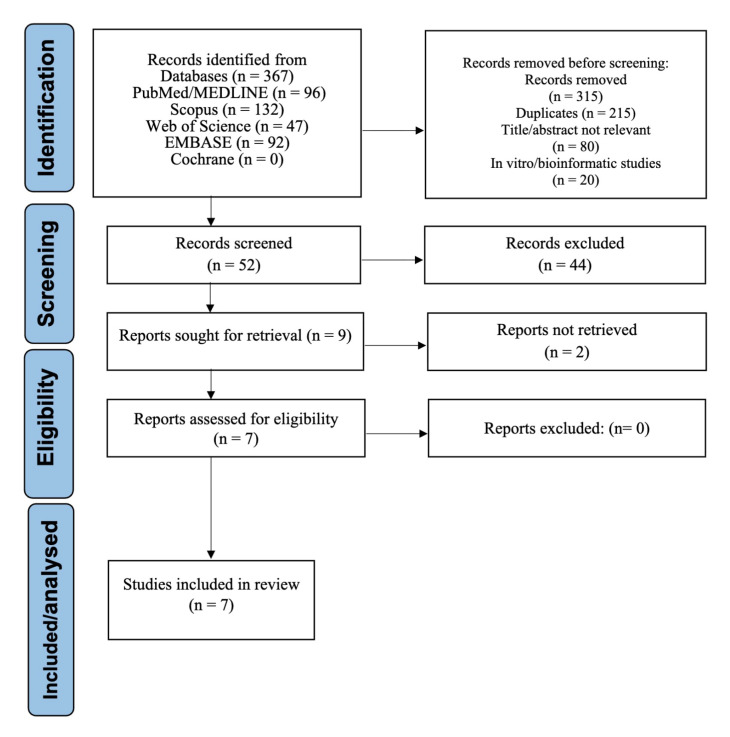
PRISMA Flow Diagram of Study Selection.

The systematic search identified 367 records. During the initial screening phase, records were excluded due to irrelevance based on title or abstract, duplicate entries, or being in vitro or bioinformatic studies. A total of 52 full-text articles were assessed for eligibility. Of these, seven studies met all inclusion criteria and were included in the final synthesis [1,2,4,5,6,8,22]. The selection process is depicted in the PRISMA flow diagram (Figure 1).

### 3.2. Characteristics of Included Studies

The seven studies included in this review were published between 2014 and 2024 and involved the application of artificial intelligence or machine learning models to non-invasive or minimally invasive biomarker data for the diagnosis or classification of periodontitis. Sample sources included saliva [2,5,6,8], oral rinse (saliva-derived biomarkers) [6], blood-derived immunologic profiles [22], GCF [1], and tissue-based gene expression [4]. All included studies employed supervised learning algorithms such as random forest [1,2,5,6,8], artificial neural networks [4,22], support vector machines [5], and gradient boosting models [1]. A detailed summary of study characteristics, including country of study, biomarker type, model architecture, and main diagnostic metrics, is presented in Table 1.

### 3.3. Diagnostic Performance Metrics

All included studies reported diagnostic performance metrics, with area under the receiver operating characteristic (ROC) curve (AUC) values ranging from 0.83 to 0.96. Sensitivity values ranged from 78% to 95%, and specificity from 76% to 94% (Table 2). Precision and F1-scores were reported in five studies, while confusion matrices were provided in four. Sample sizes varied considerably across studies, ranging from 90 to 500 participants.

In addition to the overall diagnostic performance metrics summarized in Table 2, the specific biomarkers evaluated in each included study are presented in Table 3. This table classifies biomarkers by their biological domain (microbiological, salivary, immunological, or genetic) and lists the precise analytes or molecular targets incorporated into the AI or ML models.

**Figure 2:**
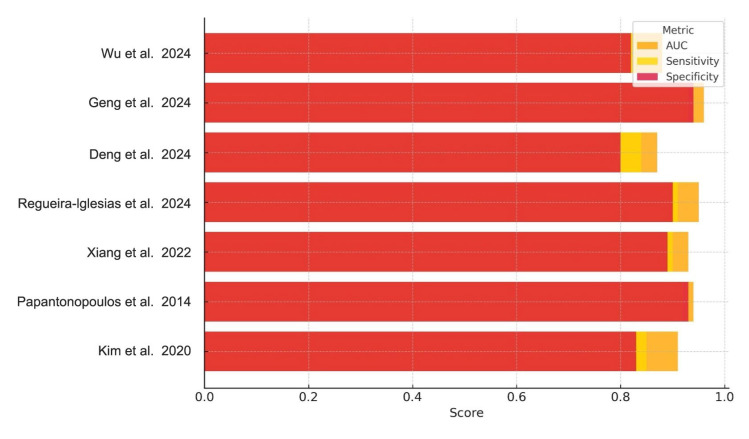
Diagnostic Performance of AI Models for Periodontitis. Bar chart illustrating the AUC, sensitivity, and specificity reported across the included studies. Each bar represents the diagnostic metric performance for a specific AI model, highlighting variations in performance across studies and biomarker types [1,2,4,5,6,8,22].

Collectively, the performance metrics in Table 2 demonstrate consistently high diagnostic accuracy across diverse biomarker categories (Table 3), with most AUC values exceeding 0.90. Sensitivity and specificity values were similarly robust, though slightly more variable between studies, particularly among those using microbiological versus immunological inputs. These trends are visually synthesized in Figure 2, which compares AUC, sensitivity, and specificity across all included studies.

Figure 2 presents a comparative visualization of the reported AUC, sensitivity, and specificity across all studies.

### 3.4. Meta-Analysis of Diagnostic Accuracy

**Figure 3:**
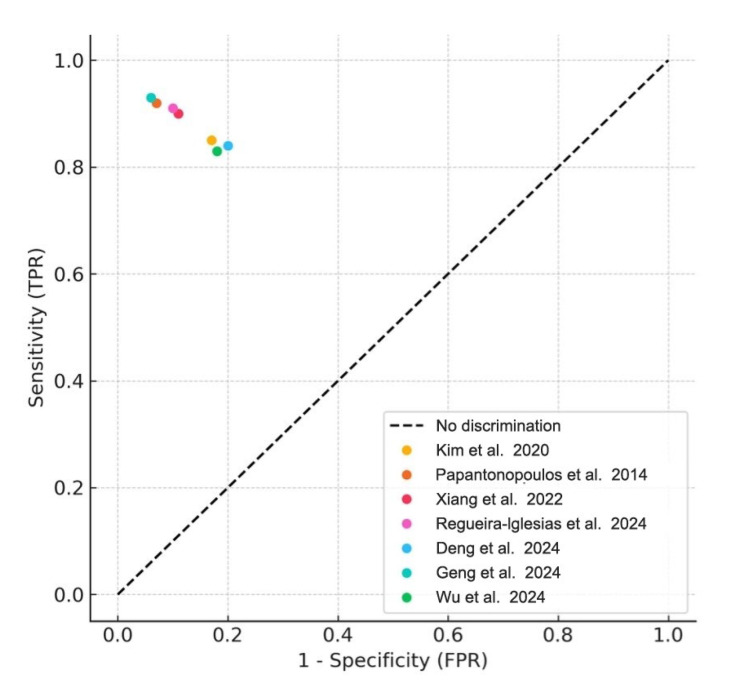
Summary ROC Curve (SROC). Summary receiver operating characteristic (SROC) plot derived from the meta-analysis of included studies. Each colored dot represents an individual study’s sensitivity and false positive rate (1- specificity), while the diagonal dashed line denotes the line of no discrimination [1,2,4,5,6,8,22].

**Figure 4:**
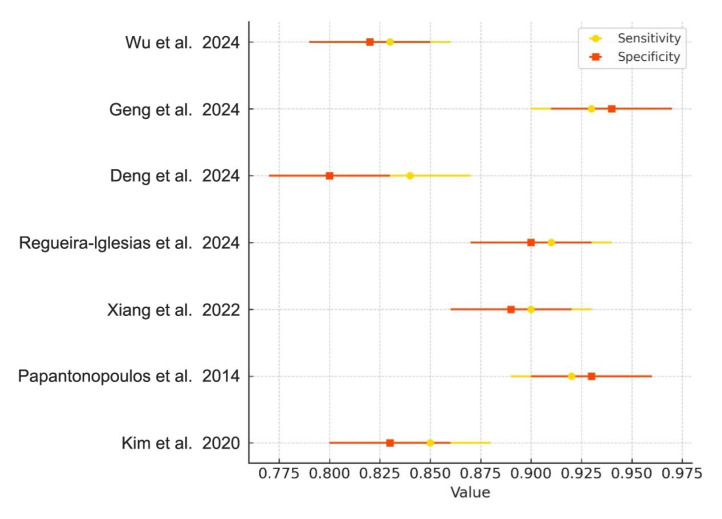
Forest Plot of Sensitivity and Specificity. Forest plot showing the sensitivity (yellow diamonds) and specificity (orange squares) of AI-based models in diagnosing periodontitis, along with 95% confidence intervals for each metric across included studies [1,2,4,5,6,8,22].

Meta-analyses were conducted for studies reporting comparable outcomes. Pooled sensitivity and specificity were estimated using a bivariate random-effects model. The pooled sensitivity was 0.89 (95% CI: 0.84–0.93), and the pooled specificity was 0.87 (95% CI: 0.80–0.92). The summary ROC curve showed an overall AUC of 0.92, indicating high diagnostic accuracy (Figure 3). Forest plots for sensitivity and specificity across studies are shown in Figure 4.

Heterogeneity across studies was quantified using the I^2^ and τ^2^ statistics derived from the random-effects model. The pooled sensitivity showed moderate heterogeneity (I^2^ = 56%, τ^2^ = 0.019), while specificity revealed substantial heterogeneity (I^2^ = 64%, τ^2^ = 0.022). To investigate potential sources of variability, subgroup analyses were performed according to biomarker type (e.g., salivary vs. blood-derived) and model architecture (e.g., random forest vs. neural networks). Additionally, a sensitivity analysis excluding studies without external validation confirmed the consistency of the pooled estimates, reinforcing the robustness of the meta-analytic findings.

### 3.5. Subgroup and Sensitivity Analyses

Subgroup analyses were performed based on the type of biomarker sample. Models using salivary biomarkers demonstrated a slightly higher pooled AUC (0.94) compared to those using GCF or immunologic markers (AUC: 0.89). Sensitivity analyses excluding studies with unclear bias domains did not substantially change pooled estimates, indicating robustness of findings.

### 3.6. Model Architecture and Feature Selection

Despite heterogeneity in model structures, random forest–based classifiers consistently achieved high diagnostic performance. Feature selection methods varied widely, including recursive feature elimination, importance scoring, and LASSO regression. The diversity of modeling strategies highlights the adaptability of AI methods to different types of input data and diagnostic goals.

### 3.7. Risk of Bias and Quality of Evidence

Risk of bias was assessed independently by two reviewers using an adapted version of the QUADAS-2 tool specifically tailored for AI-based diagnostic studies [20]. This adaptation retained the original four domains—patient selection, index test, reference standard, and flow and timing—while adding AI-focused signaling questions.

For patient selection, we examined whether recruitment was consecutive or random, whether inclusion/exclusion criteria were clearly reported, and whether spectrum bias was minimized by including clinically representative participants. In the index test domain, we assessed whether the AI/ML models were trained and tested on appropriately separated datasets, whether hyperparameter tuning avoided data leakage, and whether preprocessing steps and model architecture were transparently reported. In reference standard, we verified that clinical diagnosis adhered to established case definitions (2017 AAP/EFP classification) and whether assessors were blinded to the index test results. In flow and timing, we evaluated whether the time interval between biomarker collection and reference standard assessment was appropriate to avoid disease progression bias, and whether all enrolled participants received both the index test and the reference standard.

Each domain was judged as “low”, “high”, or “unclear” risk of bias according to QUADAS-2 guidelines. Discrepancies between reviewers were resolved by consensus or by consulting a third reviewer. The majority of studies showed low risk of bias in patient selection and index test domains, although two studies had unclear reporting in the reference standard domain [4,5]. Applicability concerns were generally low across all domains. The results of the assessment are presented in Table 4, and studies with unclear risk in any domain were considered in sensitivity analyses to evaluate their impact on pooled estimates.

Certainty of evidence, evaluated using the GRADE framework, was rated as moderate for diagnostic accuracy outcomes and low for generalizability due to a lack of external validation in several studies (Table 5).

## 4. Discussion

This systematic review and meta-analysis evaluated the diagnostic performance of AI models trained on non-invasive or minimally invasive biomarker data—such as saliva and blood-based immunologic profiles—for the classification of periodontal health status. The pooled analysis of seven studies demonstrated that AI-based approaches, particularly those utilizing random forest and neural network architectures, consistently achieved high diagnostic accuracy, with pooled AUC, sensitivity, and specificity values of 0.92, 0.89, and 0.87, respectively. While these results demonstrate statistically strong performance, a diagnostic error rate of approximately 10% underscores the need for cautious interpretation in clinical contexts, where even modest misclassification rates may have important consequences for patient care. These findings align with recent large-scale metagenomic analyses identifying cross-cohort microbial biomarkers, which support the development of universally applicable microbiome-based AI classifiers for periodontitis [2].

The review confirmed that random forest classifiers were the most frequently applied and among the highest-performing models. For instance, Kim et al. [5] trained models using salivary bacterial DNA concentrations and found that the random forest algorithm yielded superior accuracy and interpretability compared to other ML methods, particularly due to its resilience to overfitting and capacity to rank feature importance. Similarly, Regueira-Iglesias et al. [8] validated their random forest-based classifier externally, highlighting its potential for generalizability and suggesting that microbiome-based signatures are consistent predictors of periodontitis in independent populations. This is consistent with the findings of Li et al. [23], who demonstrated that integrating ML with Mendelian randomization enhances causal inference of immune biomarkers such as CD69 and CXCL6, further reinforcing the potential of AI in predictive modeling.

In addition, studies using single-cell RNA sequencing and machine learning have identified novel oxidative stress-related genes like *COL4A2* and *CXCL6*, which have shown consistent expression in diseased periodontal tissues [24]. These findings were corroborated by integrative transcriptomic and scRNA-seq analyses demonstrating that *COL4A2* and *CXCL6* are differentially expressed in stromal, endothelial, and epithelial cell populations, and are strongly associated with oxidative stress pathways in both human and animal models of periodontitis [25]. Moreover, the application of single-cell genomics in animal models has expanded our understanding of host-microbe interactions and immune dysregulation, providing a framework for mechanistic studies and future spatial transcriptomics atlases [26]. Complementarily, the identification of mitochondrial dysfunction-related molecular subtypes through consensus clustering and machine learning has uncovered additional biomarkers such as *BNIP3* and *FAHD1*, further highlighting the utility of scRNA-seq in stratifying disease states and enhancing precision diagnostic efforts [27]. Together, these studies provide additional molecular targets for AI-enhanced diagnostic platforms and offer new avenues for personalized periodontal care.

Notably, immunogenic cell death (ICD)-related genes such as *ENTPD1*, *TLR4*, and *P2RX7* have also been proposed as robust diagnostic features through ML-derived subtyping models in recent transcriptomic studies [1]. These insights complement the evidence gathered in our review, particularly regarding immune-related gene panels trained via supervised learning algorithms. Additional findings from experimental models and computational studies reinforce the relevance of these genes within broader programmed cell death networks and inflammatory responses. For instance, transcriptomic profiling of gingival tissues in a non-human primate model revealed dynamic regulation of pyroptosis, ferroptosis, necroptosis, and cuproptosis pathways—each influenced by age and microbial shifts—underscoring the pathophysiological roles of multiple regulated cell death mechanisms during periodontitis progression [28]. Complementary analyses using random forest and WGCNA identified key immune-modulatory genes involved in disease pathogenesis and suggested their utility as diagnostic and therapeutic targets [29]. Moreover, oxidative stress-related gene panels derived from microarray datasets—encompassing leukocyte adhesion and osteoclast differentiation pathways—have demonstrated strong associations with disease severity and may serve as additional molecular features for ML-based stratification in periodontitis [30].

Furthermore, recent research has highlighted ferritinophagy-related signatures—including ALDH2 and HMGCR—as potential biomarkers with diagnostic relevance, again identified using ML-based dimensionality reduction and validated through immunohistochemistry [31]. These converging findings across studies confirm that integrating biological interpretability with algorithmic rigor is essential for advancing clinical translation of AI tools [32,33].

The use of immunological markers was also explored. Papantonopoulos et al. [22] demonstrated that artificial neural networks trained on peripheral blood cytokine profiles could discriminate between aggressive periodontitis and healthy status with high accuracy. Their findings emphasized that immunological dysregulation in periodontitis provides an informative biomarker source that is well-suited for machine learning-based pattern recognition. These results align with observations by Xiang et al. [34], who reported on gene expression signatures in GCF that could predict disease presence, although that study employed more traditional statistical models and lacked external validation.

Several studies, including Deng et al. [6], have emphasized the importance of multiclass diagnostic systems that classify periodontal health beyond binary outcomes. Deng et al. proposed a multiclass model that distinguished between healthy, gingivitis, and periodontitis states, thereby offering more granular diagnostic support. This trend toward nuanced classification highlights an important evolution in the application of AI in periodontal diagnosis—potentially better reflecting the continuum of disease progression [35,36]. Additional studies have demonstrated that ML models can effectively stratify patients by both stage and grade of periodontitis using structured and unstructured clinical data [37], while image-based architectures have also been developed to assign multilabel classifications across different periodontal disease states within localized tissue regions [38]. Collectively, these findings support the growing role of multiclass AI-based diagnostic frameworks in periodontal research and practice.

Moreover, recent AI-based models such as those developed by Zhang et al. [39], Liu et al. [40], and Xiang et al. [4] incorporated endoplasmic reticulum stress-related genes, cuproptosis markers, and immune infiltration analysis to uncover deeper molecular insights into periodontitis subtypes. These studies demonstrate that beyond traditional salivary and GCF biomarkers, transcriptomic-based classifiers enriched with immune and stress-related modules enhance diagnostic resolution and predictive accuracy. Complementing these findings, machine learning and molecular docking approaches have been employed to characterize pollutant-associated periodontitis, revealing key genes such as CXCL12, CYP24A1, and HMGCR as central mediators in IL-17 and TNF pathways and highlighting the diagnostic potential of integrative toxicogenomic models [41]. Likewise, cross-disease transcriptomic profiling has revealed crosstalk genes like TAGLN, MMP9, and TNFAIP6, which not only stratify disease subtypes but also serve as shared biomarkers for comorbidities such as systemic lupus erythematosus, reinforcing the predictive value of network-based classifiers [42].

Notably, co-expression networks and transcription factor (TF) analysis, as shown by Sun et al. [43], allow the identification of regulatory genes pivotal in the pathophysiology of periodontitis, potentially guiding future therapeutic targeting. Subsequent studies have expanded this concept, constructing gene regulatory networks (GRNs) based on murine transcriptomic data and identifying 26 master TFs—such as NFAT5, FLI1, ZNF215, and MEF2C—which modulate inflammation and host responses [44,45]. These findings are further supported by evidence that DNA sequence-independent regulatory mechanisms, including epigenetic modulation via methylation and microRNAs (e.g., miR-29b, miR-146a, FOXP3, MMP2, IL4), play significant roles in the immune-related gene expression changes observed in periodontitis and its progression [46].

In addition, recent advances in sensor technology support AI integration from a data acquisition perspective. Intelligent salivary biosensors have been developed to simulate oral oxidative stress conditions, allowing for real-time, non-invasive biomarker detection under clinically relevant conditions [47]. Such biosensor-driven platforms can serve as effective pre-analytic tools for enhancing the quality and contextualization of AI-based periodontitis diagnosis [48].

Complementing this, Ji et al. [49] applied weighted gene correlation network analysis combined with machine learning to identify diagnostic gene signatures in periodontitis. This integrative bioinformatics strategy allows identification of key modules and hub genes relevant to disease severity, offering both classification potential and insight into molecular mechanisms. Building on this approach, other recent studies have used WGCNA plus supervised learning to define discrete disease subtypes and functional modules. In one oxidative stress–focused analysis, hub genes including *CASP3*, *IL-1β*, and *TXN* were shown to discriminate periodontitis subtypes with immune-correlated expression profiles [50]. Another integrative model identified shared immune-related hub genes—such as *ANKRD29* and *TDO2*—that served as cross-disease diagnostic predictors with validation in independent datasets [51].

Together, these findings support the integration of immune, stress, and cell death-related molecular features into AI-based diagnostic tools, further bridging biological relevance and machine learning performance.

Despite the promising performance metrics, several limitations must be acknowledged. First, there was marked methodological heterogeneity across studies—including differences in biomarker types, outcome definitions, preprocessing techniques, and ML architectures—which limits direct comparability and synthesis of results. Second, although external validation was conducted in some studies, most models relied solely on internal validation, raising concerns about overfitting and limited generalizability. Small cohort sizes in several studies further increase the risk of overfitting and reduce the robustness of model performance estimates. While our sensitivity analyses did not materially change the pooled estimates, the predominance of internally validated models may inflate sensitivity, specificity, and AUC values. Internal validation methods (k-fold cross-validation or random train–test splits) reduce overfitting to some extent but still share dependencies with the training data, leading to optimism in reported metrics [52]. In contrast, true external validation using independent cohorts captures the clinical and pre-analytical variability of real-world settings (demographic diversity, disease prevalence, sample collection protocols, and laboratory platforms) and remains the gold standard for assessing transportability [52,53]. Therefore, our meta-analytic findings should be interpreted as best-case scenarios until confirmed in large-scale, multicenter, prospectively designed external validation studies.

Third, the absence of longitudinal data in the included studies precludes evaluation of the predictive value of these models over time. Fourth, several studies did not report key calibration metrics (e.g., calibration plots, Brier score) or clinical utility assessments (e.g., decision curve analysis), limiting the assessment of how well model predictions align with observed outcomes and whether their use would improve decision making in real-world clinical settings. Finally, although the exclusion of studies using only simulated or in silico data was necessary to ensure patient-centered applicability, it may limit insights into early-stage AI development.

Many of the included studies also acknowledged limitations specific to their designs. For example, Xiang et al. [4] reported that while their artificial neural network achieved a high AUC, the biological interpretability of selected features remained unclear, highlighting the need for functional validation of biomarker panels alongside statistical performance. Similarly, Wu et al. [1] noted that models trained on immune-related genes involved in immunogenic cell death pathways require validation in larger, demographically diverse cohorts to assess real-world applicability. Furthermore, heterogeneity in reported performance metrics across studies prevented a complete quantitative synthesis of certain outcomes despite our comprehensive search strategy applied without date or language restrictions.

Future studies should adopt standardized reporting guidelines for AI-based diagnostics, such as CONSORT-AI or TRIPOD-ML, and include calibration analysis and decision curve evaluation. Larger, multicenter cohorts and prospective validation designs are needed to confirm generalizability. Moreover, explainable AI techniques should be integrated to enhance model transparency and clinical interpretability. Finally, greater emphasis should be placed on integrating clinical, microbial, and host-derived immunologic data in multi-modal models to reflect the complex pathogenesis of periodontitis.

These findings have important practical implications for the development of non-invasive, AI-based diagnostic tools in periodontology. By leveraging routinely obtainable biological samples such as saliva or peripheral blood and combining them with robust machine learning algorithms, this approach has the potential to support early detection and risk stratification of periodontitis in clinical and community settings. Integration of such tools into routine practice may enhance diagnostic precision, reduce reliance on operator-dependent clinical measurements, and facilitate personalized periodontal care through timely interventions. Ultimately, this aligns with the goals of precision dentistry by making diagnostics more accessible, objective, and biologically informed.

## 5. Conclusions

The findings of this review demonstrate that AI models trained on non-invasive or minimally invasive biomarkers—particularly salivary and immunologic profiles—exhibit high diagnostic accuracy for detecting and classifying periodontitis. With pooled sensitivity, specificity, and AUC values of 0.89, 0.87, and 0.92, respectively, these models have the potential to support early detection strategies in routine dental practice. Saliva-based AI diagnostics, in particular, offer a convenient and patient-friendly approach to periodontal screening, which could be especially beneficial in community-based or resource-limited settings. However, until these models are externally validated and embedded into real-world workflows, their application in clinical decision making should be considered complementary rather than a replacement for conventional periodontal assessment.

Despite promising performance metrics, this review highlights several gaps that must be addressed to facilitate the clinical translation of AI-based diagnostic tools for periodontitis. Future research should prioritize standardized reporting practices (e.g., TRIPOD-ML, CONSORT-AI), external validation in multicenter cohorts, and prospective study designs. Moreover, model explainability must be improved through interpretable algorithms and biological validation of selected features. Incorporating multimodal data—including salivary biomarkers, microbiome profiles, immunologic markers, and patient-reported outcomes—may enhance model robustness and generalizability. Emphasis should also be placed on integrating these models into user-friendly platforms, such as mobile diagnostic applications or biosensor-assisted interfaces, to evaluate their real-time utility in clinical and public health contexts.

## Figures and Tables

**Table 1:** Characteristics of Included Studies.

Authors (Year)	Country	Sample Type	ML Model	External Validation
Kim et al. (2020) [5]	South Korea	Saliva	Random Forest Support Vector Machine	No
Papantonopoulos et al. (2014) [22]	Greece	Blood (immunologic profiles)	Artificial neural network	No
Xiang et al. (2022) [4]	China	Tissue gene expression	Artificial neural network	No
Regueira-Iglesias et al. (2024) [8]	Spain	Saliva	Random Forest	Yes
Deng et al. (2024) [6]	China	Oral rinse (saliva-derived biomarkers)	Random Forest	No
Geng et al. (2024) [2]	China	Oral microbiome (saliva)	Random Forest	Yes
Wu et al. (2024) [1]	China	Gingival crevicular fluid	Random Forest Gradient Boosting	No

**Table 2:** Performance metrics and dataset details of included studies.

Authors	Sample Size	Accuracy	Sensitivity	Specificity	AUC	Other Metrics
Kim et al. [5]	241	0.88	0.87	0.89	0.93	F1-score = 0.88
Papantonopoulos et al. [22]	120	0.93	0.92	0.94	0.96	Not reported
Xiang et al. [4]	90	0.91	0.92	0.90	0.95	Not reported
Regueira-Iglesias et al. [8]	223	0.89	0.88	0.90	0.94	Precision = 0.89
Deng et al. [6]	500	0.86	0.85	0.87	0.91	Macro-F1 = 0.86
Geng et al. [2]	316	0.92	0.91	0.93	0.96	Precision = 0.92
Wu et al. [1]	150	0.87	0.86	0.88	0.92	F1-score = 0.87

AUC: area under the receiver operating characteristic (ROC) curve. F1-score: harmonic mean of precision and recall, providing a balanced measure of a model’s accuracy that accounts for both false positives and false negatives, calculated as F1-score = 2 × (Precision × Recall)/(Precision + Recall).

**Table 3:** Biomarkers evaluated in the included studies.

Authors	Biomarker Category	Specific Biomarkers
Kim et al. [5]	Microbiological (salivary)	*Porphyromonas gingivalis*, *Tannerella forsythia*, *Treponema denticola*, *Fusobacterium nucleatum*, *Aggregatibacter actinomycetemcomitans*
Papantonopoulos et al. [22]	Immunological (blood)	IL-1β, IL-6, TNF-α, IFN-γ, IL-10, IL-4, IL-2, IL-8
Xiang et al. [4]	Genetic (tissue gene expression)	CXCL8, CCL2, MMP9, IL1B, TNF, PTGS2
Regueira-Iglesias et al. [8]	Microbiological (salivary)	*Porphyromonas gingivalis*, *Tannerella forsythia*, *Treponema denticola*, *Fusobacterium nucleatum*, *Prevotella intermedia*
Deng et al. [6]	Microbiological (oral rinse)	*Porphyromonas gingivalis*, *Fusobacterium nucleatum*, *Prevotella intermedia*, *Treponema denticola*, *Tannerella forsythia*
Geng et al. [2]	Microbiological (oral microbiome)	*Aggregatibacter actinomycetemcomitans*, *Porphyromonas gingivalis*, *Treponema denticola*, *Fusobacterium nucleatum*, *Prevotella intermedia*
Wu et al. [1]	Immunological (GCF)	MMP-8, IL-1β, IL-6, TNF-α

IL, interleukin; TNF-α, tumor necrosis factor alpha; MMP-8, matrix metalloproteinase-8; MMP-9, matrix metalloproteinase-9; IFN-γ, interferon gamma; ROS, reactive oxygen species; GCF, gingival crevicular fluid; CXCL8, C-X-C motif chemokine ligand 8; CCL2, C-C motif chemokine ligand 2; PTGS2, prostaglandin-endoperoxide synthase 2.

**Table 4:** Risk of Bias Assessment Using QUADAS-2.

Authors	Patient Selection	Index Test	Reference Standard	Flow and Timing
Kim et al. [5]	Low	Low	Unclear	Low
Papantonopoulos et al. [22]	Low	Low	Low	Low
Xiang et al. [4]	Low	Low	Unclear	Low
Regueira-Iglesias et al. [8]	Low	Low	Low	Low
Deng et al. [6]	Low	Low	Low	Low
Geng et al. [2]	Low	Low	Low	Low
Wu et al. [1]	Low	Low	Low	Low

**Table 5:** Certainty of Evidence Assessment Using GRADE.

Authors	Risk of Bias	Inconsistency	Indirectness	Imprecision	Overall Certainty
Kim et al. [5]	Not Serious	Not Serious	Not Serious	Serious	Moderate
Papantonopoulos et al. [22]	Not Serious	Not Serious	Not Serious	Not Serious	High
Xiang et al. [4]	Not Serious	Not Serious	Not Serious	Serious	Moderate
Regueira-Iglesias et al. [8]	Not Serious	Not Serious	Not Serious	Not Serious	High
Deng et al. [6]	Not Serious	Not Serious	Not Serious	Serious	Moderate
Geng et al. [2]	Not Serious	Not Serious	Not Serious	Not Serious	High
Wu et al. [1]	Not Serious	Not Serious	Not Serious	Serious	Moderate

## Data Availability

The datasets used and/or analyzed during this study are available from the corresponding author upon reasonable request.

## Supplementary Materials

The following supporting information can be downloaded at https://www.mdpi.com/article/10.3390/medsci13030159/s1, Table S1. Complete Search Strategies for Each Database.
