# Supplementary material for: Artificial Intelligence Models for Diagnosis of Periodontitis Using Non-Invasive Biological Markers: A Systematic Review and Meta-Analysis of Patient-Based Studies
Source: Med Sci (Basel). 2025 Sep 1;13(3):159. doi: 10.3390/medsci13030159 (PMC12452651; doi:10.3390/medsci13030159)
Supplement: Supplementary file 1 [file medsci-13-00159-s001.zip › medsci-3830199- Supplementary Materials.pdf]

Supplementary Table S1. Complete Search Strategies for Each Database

The following table details the complete search strategies used for each database, including all search terms, Medical Subject Headings (MeSH), keywords, Boolean operators, and date of last search. The search strategies were adapted to the syntax and indexing requirements of each database. No language or date restrictions were applied.

Database	Search Strategy	Date Last Searched
PubMed/MEDLINE	("periodontitis"[MeSH Terms] OR "periodontal disease"[All Fields]) AND ("artificial intelligence"[MeSH Terms] OR "machine learning"[All Fields]) AND ("saliva"[MeSH Terms] OR "biological markers"[MeSH Terms] OR "gingival crevicular fluid"[All Fields] OR "microbiome"[MeSH Terms])	June 30, 2025
Scopus	TITLE-ABS-KEY("periodontitis" OR "periodontal disease") AND TITLE-ABS-KEY("artificial intelligence" OR "machine learning") AND TITLE-ABS-KEY("saliva" OR "biological marker*" OR "gingival crevicular fluid" OR "microbiome")	June 30, 2025
Web of Science	TS=("periodontitis" OR "periodontal disease") AND TS=("artificial intelligence" OR "machine learning") AND TS=("saliva" OR "biological marker*" OR "gingival crevicular fluid" OR "microbiome")	June 30, 2025
EMBASE	('periodontitis'/exp OR 'periodontal disease') AND ('artificial intelligence'/exp OR 'machine learning') AND ('saliva'/exp OR 'biological marker'/exp OR 'gingival crevicular fluid' OR 'microbiome'/exp)	June 30, 2025

Cochrane CENTRAL	("periodontitis" OR "periodontal disease") AND ("artificial intelligence" OR "machine learning") AND ("saliva" OR "biological marker*" OR "gingival crevicular fluid" OR "microbiome")	June 30, 2025
